# Mitogenomic Architecture of Atlantic Emperor *Lethrinus atlanticus* (Actinopterygii: Spariformes): Insights into the Lineage Diversification in Atlantic Ocean

**DOI:** 10.3390/ijms251910700

**Published:** 2024-10-04

**Authors:** Shantanu Kundu, Hye-Eun Kang, Yunji Go, Gyurim Bang, Yengju Jang, Hsu Htoo, Sarifah Aini, Hyun-Woo Kim

**Affiliations:** 1 Ocean and Fisheries Development International Cooperation Institute, College of Fisheries Science, Pukyong National University, Busan 48513, Republic of Korea; 2 International Graduate Program of Fisheries Science, College of Fisheries Science, Pukyong National University, Busan 48513, Republic of Korea; 3 Institute of Marine Life Science, Pukyong National University, Busan 48513, Republic of Korea; 4 Industry 4.0 Convergence Bionics Engineering, Pukyong National University, Busan 48513, Republic of Korea; 5 Department of Marine Biology, Pukyong National University, Busan 48513, Republic of Korea; 6 Research Center for Marine Integrated Bionics Technology, Pukyong National University, Busan 48513, Republic of Korea; 7 Department of Biology, Faculty of Science and Technology, Airlangga University, Surabaya 60115, Indonesia

**Keywords:** marine fish, mitogenome, phylogeny, lineage diversification, evolution, oceanology

## Abstract

The evolutionary history of emperors, particularly in the Atlantic and Indo-West Pacific Oceans, remains largely unmapped. This study explores the maternal lineage evolution of Lethrinids by examining the complete mitogenome of *Lethrinus atlanticus*, which is endemic to the Eastern Atlantic Ocean. Utilizing advanced next-generation sequencing, we found that the mitogenome spans 16,789 base pairs and encompasses 37 genes, including 13 protein-coding genes (PCGs), two ribosomal RNAs, 22 transfer RNAs, and an AT-rich control region (CR). Our analysis indicates a preference for AT base pairs in the *L. atlanticus* mitogenome (53.10%). Most PCGs begin with the ATG codon, except for *COI*, which starts with GTG. Relative synonymous codon usage reveals high frequencies for alanine, leucine, proline, serine, and threonine. The ratio of nonsynonymous to synonymous substitutions suggests strong negative selection across all PCGs in *Lethrinus* species. Most transfer RNAs exhibit typical cloverleaf structures, with the exception of *tRNA-serine* (GCT), which lacks a dihydrouracil stem. Comparative analysis of conserved sequence blocks across the CRs of three *Lethrinus* species shows notable differences in length and nucleotide composition. Phylogenetic analysis using concatenated PCGs clearly distinguishes all *Lethrinus* species, including *L. atlanticus*, and sheds light on the evolutionary relationships among Spariformes species. The estimated divergence time of approximately 20.67 million years between *L. atlanticus* and its Indo-West Pacific relatives provides insights into their historical separation and colonization during the late Oligocene. The distribution of Lethrinids may be influenced by ocean currents and ecological factors, potentially leading to their speciation across the Eastern Atlantic and Indo-West Pacific. This study enhances our understanding of the genetic diversity and phylogenetic relationships within *Lethrinus* species. Further exploration of other emperor fish mitogenomes and comprehensive genomic data could provide vital insights into their genetic makeup, evolutionary history, and environmental adaptability in marine ecosystems globally.

## 1. Introduction

The mitochondrial genome, typically spanning 16–17 kbp, is highly conserved across eukaryotic organisms due to its crucial role in cellular respiration [1]. However, its relatively high mutation rate, resulting from fewer efficient mitochondrial DNA (mtDNA) repair mechanisms compared to nuclear DNA (nDNA), makes it particularly valuable for studying evolutionary relationships among organisms. Biologists analyze and compare the structure and variations of mitochondrial genomes among different species to reconstruct evolutionary histories and trace lineage diversification in vertebrates over time and across various regions [2]. In addition to cladistic analyses, understanding the genetic makeup and arrangement of genes within the mitogenome, encompassing 13 PCGs (protein-coding genes), 2 rRNAs (ribosomal RNA genes), 22 tRNAs (transfer RNA genes), and a non-coding AT-rich CR (control region), is essential for deciphering the roles of these genes [3]. To date, over 15,000 Actinopterygii mitogenomes have been sequenced globally and added to the GenBank database (https://www.ncbi.nlm.nih.gov/, accessed on 20 May 2024). This dataset is enriched by the addition of 103 mitogenomes from Spariformes bony fishes within the class Actinopteri, although it predominantly includes contributions from the families Sparidae and Nemipteridae. This highlights the need for further mitogenomic research on the lesser-studied families Lethrinidae and Centracanthidae within this group.

Emperor fishes and large-eye breams (family Lethrinidae) belong to the suborder Percoidei and are classified within the superfamily Sparoidea, alongside Nemipteridae, Sparidae, and Centracanthidae [4]. The evolutionary relationships among these families were later reassessed based on morphological characteristics, leading to the subdivision of Lethrinidae into the subfamilies Lethrininae and Monotaxinae, which are distinguished by head scalation patterns and the counts of dorsal and anal fin rays [5]. Members of Lethrinidae are characterized by strong jaws with unique dentition patterns and are demersal fish, exhibiting three distinct trophic modes. Low-bodied species with conical lateral teeth primarily feed on mobile prey, such as fish and certain crustaceans. High-bodied species with molariform teeth typically consume slow-moving invertebrates like molluscs, sea urchins, and some crustaceans. Other high-bodied species with conical teeth mainly ingest slow-moving invertebrates, including soft-shelled or small hard-shelled invertebrates [6,7].

To date, 46 valid species within the family Lethrinidae have been described globally, as reported in Eschmeyer’s Catalog of Fishes database [8]. Most of these species are distributed in the Indo-West Pacific Ocean, although the Atlantic Emperor, *Lethrinus atlanticus*, enigmatically resides in the eastern Atlantic, suggesting the need for further research into its lineage diversification over time and space. The Lethrinids, particularly the genus *Lethrinus*, include many species vital to commercial and subsistence fisheries worldwide [5]. Therefore, understanding their biology and ecology at the molecular level is crucial for sustainable fisheries management, necessitating accurate species identification and insights into their evolutionary history [9]. To elucidate genetic diversity, population structure, evolution, and the potential existence of cryptic diversity, morphometric and cranial characters, along with genetic evaluations using various mitochondrial genes, have been conducted on Lethrinids [7,10,11,12,13]. In addition to mitochondrial markers, microsatellite markers have been employed to investigate the genetic diversity of these reef-associated fishes [14]. Progress in molecular methodologies has facilitated the production of complete mitochondrial genome sequences for emperors, assisting in illuminating the phylogenetic placement of tetraodontiform fishes within the broader group of higher teleosts [15,16]. Beyond molecular research, ecological studies have assessed the impacts of anthropogenic factors on *Lethrinus* species for conservation management and sustainable use [17,18,19]. Furthermore, the reproductive biology, demographic structure, and stock status of emperor fishes have been investigated to inform their effective management [20,21].

To date, the mitogenomes of four species within the family Lethrinidae have been sequenced worldwide. These mitogenomes represent two subfamilies: Monotaxinae, (*Monotaxis grandoculis* and *Gnathodentex aureolineatus*) and Lethrininae (*Lethrinus obsoletus* and *Lethrinus laticaudis*) [16,22,23]. Therefore, mitogenomic evaluations of Lethrinids have been limited to the Red and Indo-West Pacific Ocean. To enhance taxonomic coverage and understand the evolutionary relationships within the Lethrinidae family, the present study aims to sequence the mitogenome of *L. atlanticus* from the Atlantic Ocean. This study has the following objectives: (i) to compare the genetic traits of mitochondrial genes in *L. atlanticus* and its congeners, (ii) to analyze the structures and variations of tRNAs and CRs, (iii) to assess the maternal evolutionary relationships of Lethrinids with other Spariformes, and (iv) to determine the divergence time and diversification patterns of Lethrinids in the Indo-West Pacific and Atlantic marine biomes. Overall, integrating mitogenomic traits and evolutionary insights into conservation plans will enable more effective management of the Atlantic Emperor, helping to preserve its genetic integrity and ensure long-term viability in the face of environmental and anthropogenic pressures. Such studies will also help infer the matrilineal phylogeny of other Spariformes species and provide new insights into the functions of different mitochondrial genes in the evolution of these enigmatic reef-associated fish across marine environments.

## 2. Results and Discussion

### 2.1. Mitogenome Structure and Organization

**Figure 1:**
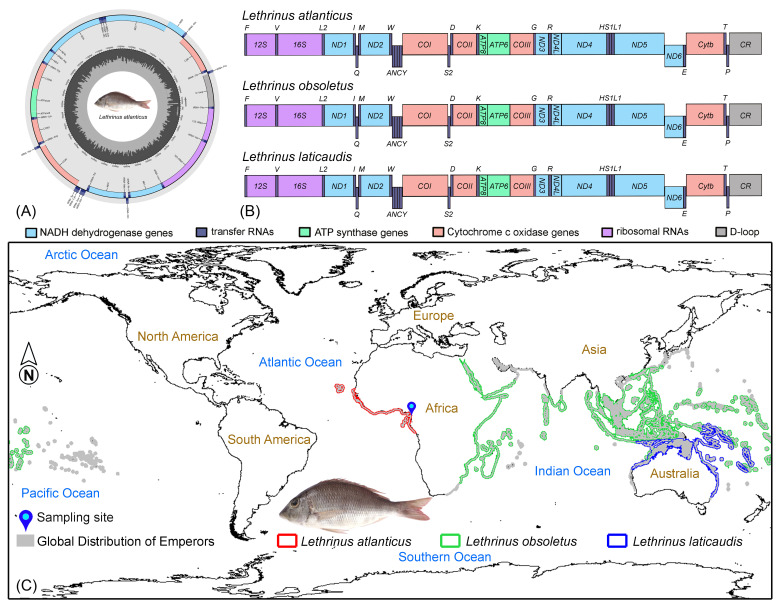
(**A**) The spherical representation of the mitogenome of *L. atlanticus* is provided, annotated using MitoAnnotator. Various color arcs delineate the occurrence of PCGs, tRNAs, rRNAs, and CR. (**B**) The linearized representation of the complete mitogenomes showcases the resemblance with the ancestral vertebrate gene order across the three *Lethrinus* species. (**C**) Global distribution pattern of *L. atlanticus* and other *Lethrinus* species within the marine environment. The sampling locality of *L. atlanticus* is denoted by a blue pin.

The mitogenome of *L. atlanticus* (16,789 bp) was also determined in the present study (GenBank Accession No. OQ420716) (Figure 1A). The complete length of *L. atlanticus* is high in comparison with the other two species, *L. laticaudis* (16,758 bp) and *L. obsoletus* (16,779 bp). All three *Lethrinus* species mitogenomes constituted 13 PCGs (12 in the positive strand and 1 in the negative strand), 22 tRNAs (14 in the positive strand and 8 in the negative strand), as well as 2 rRNAs and an AT-rich CR in the positive strand (Table 1, Figure 1B). The mitogenome of *L. atlanticus* is AT biased (53.10%), with 27.20% A, 25.90% T, 17.02% G, and 29.88% C. Similarly, AT bias (52.61% and 52.01%) was also observed in other species, *L. laticaudis* and *L. obsoletus*, respectively. In the *L. atlanticus* mitogenome, the AT skew and GC skew were 0.024 and −0.274. The present analysis also showed similar skewness in other *Lethrinus* species, with 0.026 AT skew and −0.279 GC skew in *L. laticaudis* as well as 0.029 AT skew and −0.281 GC skew in *L. obsoletus* (Table 2). A total of 11 intergenic spacers with a total length of 76 bp and 6 overlapping regions with a total length of 14 bp were observed in *L. atlanticus* mitogenome (Supplementary Table S1). The distribution of the intergenic spacers is high in the other two species, *L. laticaudis* (14 with 101 bp total length) and *L. obsoletus* (13 with 172 bp total length). The distribution of overlapping regions is almost the same in *L. atlanticus* and *L. obsoletus*, but is different in *L. laticaudis* (11 with 23 bp total length). In *L. obsoletus*, the longest intergenic spacer (96 bp) was found between *tRNA-Val (V)* and *16S rRNA*. The most extensive overlapping region (7 bp) was consistently observed between *ND4L* and *ND4* in the mitogenomes of all three *Lethrinus* species (Supplementary Table S1). The genetic differences observed within the complete mitochondrial genomes of *Lethrinus* species offer valuable perspectives into their energy metabolism and evolutionary patterns, aligning with similar results in other fish species [24,25]. This research provides a vital understanding of the structural features of *Lethrinus* mitogenomes, thereby enriching our comprehension of the functions encoded by mitochondrial genes.

### 2.2. Protein-Coding Genes

The entire length of *L. atlanticus* PCGs was 11,425 bp, which is 68.05% of the complete mitogenome. The total length of PCGs was similar in *L. atlanticus* and *L. obsoletus*, but lower (11,423 bp) in *L. laticaudis*. All three *Lethrinus* species PCGs were AT biased (50.95% to 52.48%). The AT and GC skews ranged from −0.061 (*L. laticaudis*) to −0.056 (*L. obsoletus*) and −0.313 (*L. obsoletus*) to −0.308 (*L. atlanticus*). Most of the PCGs are initiated with an ATG start codon excluding the *COI* gene, GTG in *L. atlanticus* and *L. obsoletus*, but ATT in *L. laticaudis*. The PCGs of *L. atlanticus* are terminated by TAG (*ND1* and *ND6*), TAA (*ATP8*, *COI*, *ND4L*, and *ND5*), and incomplete stop codons (*ATP6*, *COII*, *COIII*, *ND2*, *ND3*, *ND4*, and *CYTB*). Almost all of the PCGs of *L. laticaudis* terminated with the TAA stop codon, except *ND3* and *ND6* by TAG, and the other PCGs with an incomplete stop codon. In *L. obsoletus*, five PCGs (*ATP8*, *COI*, *ND1*, *ND4L*, and *ND6*) were terminated with TAA, *ND5* was terminated with TAG, and others ended with an incomplete stop codon (Supplementary Table S2). Incomplete termination codons like these may be potentially supplemented with TAA during RNA processing, as proposed in earlier studies [26]. As seen in various fish species, the distinguished genetic differences may potentially prompt distinct selection pressures among PCGs [27,28]. These PCGs are crucial for ATP synthesis, oxidative phosphorylation, and the encryption of proteins demanded in electron transport pathways. Consequently, the inclusion of complete mitochondrial genomes from various emperors could aid in exploring distinctions in gene expression and energy operation.

### 2.3. Relative Synonymous Codon Usage and Substitutions Pattern

**Figure 2:**
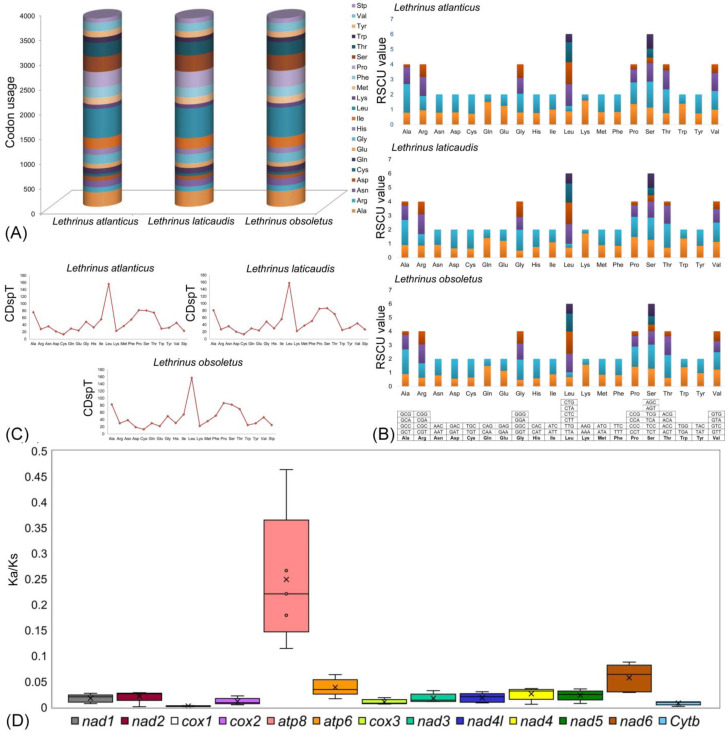
(**A**) The codon usage abundance in mitochondrial genomes of *Lethrinus* species, including *L. atlanticus*; (**B**) a comparative analysis of relative synonymous codon usage (RSCU) across *Lethrinus* species, where collective RSCU values are plotted on the y-axis against codons for each amino acid on the x-axis; (**C**) codon distribution per thousand codon values for all amino acids in the mitochondrial genomes of the three *Lethrinus* species; and (**D**) a box plot illustrating the pairwise deviation of the nonsynonymous (Ka)/synonymous (Ks) ratio for PCGs in the mitogenomes of the three *Lethrinus* species.

Codons representing individual amino acids were observed to remain conserved across all PCGs in the compared *Lethrinus* species. An investigation of relative synonymous codon usage (RSCU) indicated a predominant occurrence of alanine, proline, serine, leucine, and threonine within the PCGs of *L. atlanticus*, while cysteine, aspartic acid, glutamic acid, lysine, and tryptophan were less prevalent (Figure 2A). Similar distributions of amino acid richness were noted in the other two *Lethrinus* species (Supplementary Tables S3 and S4). Notably, the RSCU analysis disclosed a noteworthy reduction (≤0.5) in the occurrence of specific codons—TTG for leucine, TCG for serine, CCG for proline, ACG for threonine, GCG for alanine, CAG for glutamic acid, and AAG for lysine—in *L. atlanticus* (Figure 2B, Supplementary Table S3). In comparison with *L. atlanticus*, *L. laticaudis* shows a similar type of significant reduction in the occurrence of the codons in proline, threonine, alanine, and an additional GGT codon in glycine; however, in *L. obsoletus* the decreasing trends in the frequency of the codons were observed only in leucine, serine, proline, threonine, alanine, and lysine (Figure 2B, Supplementary Table S3). Codon distribution per thousand codons (CDspT) values for all the amino acids exhibited identical results ranging from 12.884 in cysteine (*L. obsoletus*) to 157.99 in leucine (*L. laticaudis*) (Figure 2C).

The Darwinian selection hypothesis remains integral in explaining the evolutionary kinetics of genes experiencing positive selection, thereby acting a fundamental function in species differentiation [29,30]. Scrutiny of synonymous (Ks) and Nonsynonymous (Ka) substitution rates within PCGs furnishes insights into the assortment and adaptive molecular evolution in craniates, including fish [31]. The Ka/Ks ratio aids as a well-established metric for assessing selective pressure and evolutionary associations at the molecular level, and is pertinent across both uniform and diverse species [32]. In this research, we explored the evolutionary rates among homologous gene pairs by computing Ka/Ks substitutions for *L. laticaudis* and associating them with those of *L. obsoletus*. The Ka/Ks ratio varied from 0.0030 ± 0 in COI to 0.2663 ± 0.179 in *ATP8*, following this sequence: *COI* < *CYTB* < *COIII* < *COII* < *ND3* < *ND1* < *ND4L* < *ND2* < *ND5* < *ND4* < *ATP6* < *ND6* < *ATP8* (Figure 2D). Most PCGs displayed Ka/Ks values below ‘1’, representing strong negative selection within the examined *Lethrinus* species, signifying that those mutations were predominantly substituted by synonymous substitutions (Supplementary Table S5). This opinion highlights the role of natural selection in eliminating deleterious alterations with negative selective effects, consistent with broader patterns detected in other vertebrates [31,33]. Therefore, the comparative investigation of Ka/Ks across *Lethrinus* species’ mitochondrial genomes offers insights into the shades of natural selection influencing species’ evolutionary trajectories. This examination contributes to unraveling the complicated relationship between mutations and selective pressures, clarifying their collective impact on protein evolution. The interplay of strong negative selection in PCGs significantly enhances the evolutionary adaptability of *Lethrinus* species, promoting genetic stability and resilience while facilitating the emergence of new species through ecological colonization. By preserving essential functions and eliminating deleterious mutations, this selection process allows for specialized adaptations to diverse marine environments. Understanding the specific mechanisms of negative selection in fish species can provide crucial insights into their evolutionary history and inform conservation strategies, particularly in the context of rapidly changing environmental conditions.

### 2.4. Ribosomal RNA and Transfer RNA Genes

In comparison with three *Lethrinus* species, the total length of rRNAs ranged from 2664 bp (*L. obsoletus*) to 2761 bp (*L. laticaudis*). The rRNA genes are AT-biased ranged from 52.27% (*L. atlanticus*) to 53.04% (*L. obsoletus*). The AT and GC skews ranged from 0.179 (*L. laticaudis*) to 0.184 (*L. atlanticus*) and from −0.122 (*L. atlanticus*) to −0.111 (*L. laticaudis*) respectively. The studied species (*L. atlanticus*) contained 22 tRNA genes in their respective mitogenome. In *L. atlanticus*, the total length of tRNAs was 1562 bp, which shared 9.3% of the complete mitochondrial genome. In comparison with other *Lethrinus* species, the entire length of tRNA ranged from 1552 bp (*L. laticaudis*) to 1563 bp (*L. obsoletus*). The *Lethrinus* species tRNA genes were AT biased, ranging from 54% (*L. obsoletus*) to 54.9% (*L. laticaudis*) and AT skew ranged from −0.007 (*L. laticaudis*) to 0.012 (*L. obsoletus*). In *L. atlanticus*, the predominant transfer RNAs exhibit conventional cloverleaf secondary structures, except for tRNA-serine (GCT), which lacks a dihydrouracil (DHU) stem (Supplementary Figure S1). Wobble base pairings were detected in 14 tRNAs of *Lethrinus* atlanticus, with the most prominent occurrences observed in *trnA* and *trnE*. Specifically, wobble base pairings were identified in the DHU stem of six tRNAs, the TψC stem of four tRNAs, the anticodon stem of five tRNAs, and the acceptor stem of ten tRNAs (Supplementary Figure S1). Wobble base pairing, a significant aspect of RNA structure, frequently substitutes for GC or AT base pairs because of its thermodynamic stability. Homogeneity in the anticodons of all 22 tRNAs was observed across all *Lethrinus* species (Supplementary Table S6). These traits play pivotal roles in various biological processes [26,34]. RNA-binding proteins selectively recognize and bind to distinct G-U sites by discerning chemical features that set them apart from Watson–Crick and other mismatched pairs [35]. Additionally, the rearrangement of tRNAs and the presence of substantial length heteroplasmy in the cluster of five tRNAs (WANCY region) offer insights into the evolution of mitogenomes [36,37]. Therefore, a comprehensive exploration of tRNAs is essential for understanding the structural and functional attributes of fish mitogenomes [2,32].

### 2.5. Characteristics of Control Region

**Figure 3:**
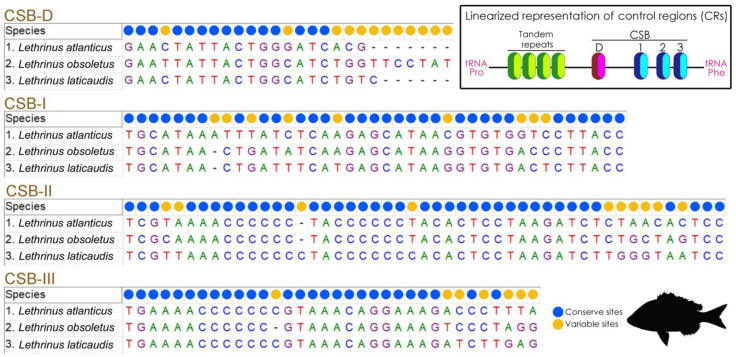
Schematic evaluation of the nucleotide composition and length of different conserved domains among *L. atlanticus* and two other *Lethrinus* species’ CR. Variable and conserved nucleotides are indicated by blue and yellow circles, respectively, with gaps represented by a dash symbol. The linearized representation of the CR is showed in inset.

The CR of *L. atlanticus* spans 983 bp, comprising 5.86% of the complete mitochondrial genome. Compared to other *Lethrinus* species, the length of the CR ranges from 944 bp (*L. laticaudis*) to 983 bp (*L. atlanticus*). The CRs exhibit an AT bias, ranging from 59.9% (*L. obsoletus*) to 61.85% (*L. atlanticus*), with AT skews ranging from −0.009 (*L. obsoletus*) to 0.010 (*L. atlanticus*). A thorough examination of the mitogenomes of three *Lethrinus* species, informed by previous studies [2], reveals the presence of four conserved sequence blocks (CSBs)—CSB-D, CSB-I, CSB-II, and CSB-III in the CR of *L. atlanticus* and the other two *Lethrinus* species. This pattern is uniform with findings in other teleost mitochondrial genomes [28,32]. Comparative analyses reveal significant nucleotide variability within the conserved blocks. CSB-II is the longest at 49 bp, while CSB-I, CSB-III, and CSB-D are 41 bp, 34 bp, and 27 bp long, respectively (Figure 3). Specifically, CSB-D shows notable nucleotide variability with 12 variable base pairs (44.44%), whereas CSB-I, CSB-II, and CSB-III are predominantly conserved, with 78.05%, 79.6%, and 79.42% conserved sites, respectively (Figure 3). Furthermore, the extended termination-associated sequence (ETAS) region, characterized by a plethora of repeats, appears as the utmost dynamically variable part within the CR. It is distinguished by exact motifs that give rise to stable hairpin loops. However, unlike other fish species, the CR of *Lethrinus* species lacks tandem repeats in the ETAS region. Beyond these conserved elements, the CRs of *Lethrinus* species contain extremely polymorphic base pairs, which serve as a robust marker for species differentiation and the elucidation of phylogeography in fish, phenomena extensively observed in other fish species [38,39]. Moreover, the intricate mechanisms in the CR, such as genetic arrangement through double replications, dimer-mitogenomes, as well as random and non-random loss, significantly lead to understanding the structural variety of mitogenomes and the complexities inherent in mitogenome evolution.

### 2.6. Major Phylogenetic Relationship of Spariformes

**Figure 4:**
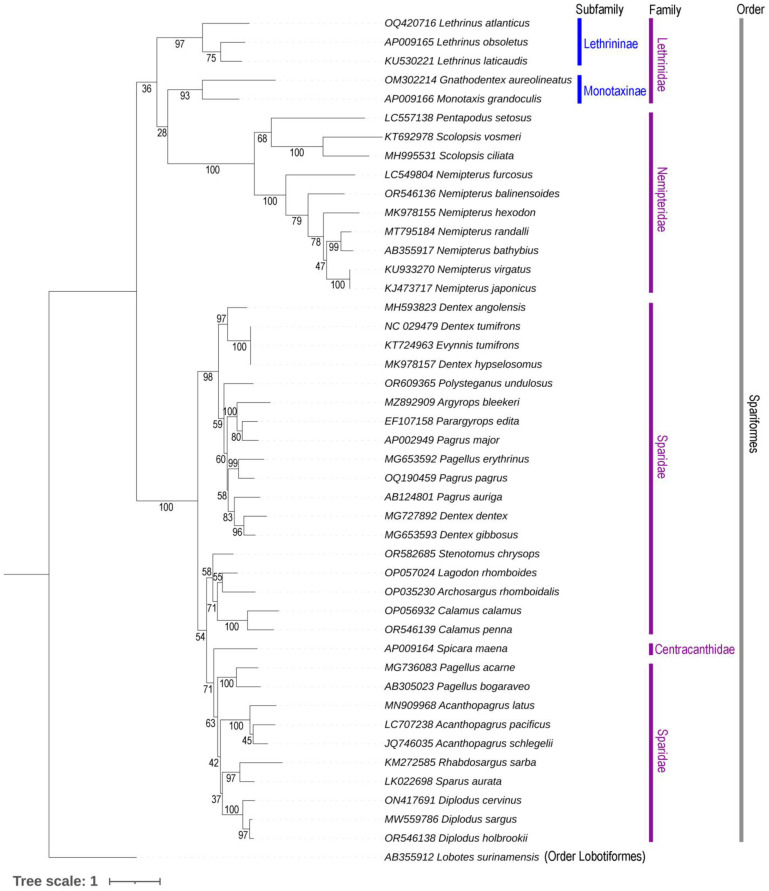
Maximum Likelihood (ML) topology built using concatenated 13 PCGs, effectively distinguishing *L. atlanticus* from other *Lethrinus* species. The cladogram also offers perceptions into the evolutionary relations across diverse taxonomic levels (subfamily and family) within the Spariformes order. ML bootstrap supports are annotated at each node (black values), indicating the statistical support for individual branch in the topology.

The ML and BA topologies, constructed using concatenated PCGs, distinctly separate all Spariformes species, elucidating their evolutionary relationships (Figure 4, Supplementary Figure S2). Species from various taxonomic lineages, spanning both family and subfamily levels, display distinct monophyletic clustering patterns. Three *Lethrinus* species (*L. atlanticus*, *L. laticaudis*, and *L. obsoletus*) within the subfamily Lethrininae display a cohesive clustering pattern and a sister relationship with other Monotaxinae subfamily members, aligning with previous evolutionary hypotheses on Lethrinidae [40,41]. Surprisingly, a single species from the family Centracanthidae, *Spicara maena*, clustered with the Sparidae clade, warrants further investigation in future analyses considering multiple mitogenomic sequences of Centracanthus and *Spicara* species. In this major matrilineal phylogenetic relationship of Spariformes, the topologies indicate that species within the family Lethrinidae are evolutionarily close to those in the family Nemipteridae (threadfin breams) (Figure 4, Supplementary Figure S2).

Nevertheless, this investigation highlights the usefulness of complete mitogenomes in discerning and explaining the evolutionary associations among Spariformes species, a consistency observed in other Teleostei studies [28,32]. Extensive genomic datasets provide a worthwhile understanding of time-calibrated phylogeny, adaptation to varying salinity levels, and the process of speciation [42]. However, previous evolutionary interpretations of Lethrinidae have predominantly relied on partial mitochondrial or nuclear gene sequences [7,40,43,44]. Acknowledging the vital role of genomic data in conservation genetics and fisheries management [45], this study recommends the generation of further large-scale genomic datasets focusing on this group, particularly members of the Lethrinidae and Centracanthidae families. Such efforts will enhance our understanding of the evolution, diversification, and adaptation of these reef-associated fishes in marine environments. In addition to the mitogenomic exploration of Spariformes, employing a large-scale phylogenomic approach will provide a more accurate evolutionary depiction of these lineages within marine ecosystems [46].

### 2.7. Lineage Diversification of Lethrinidae in Atlantic

**Figure 5:**
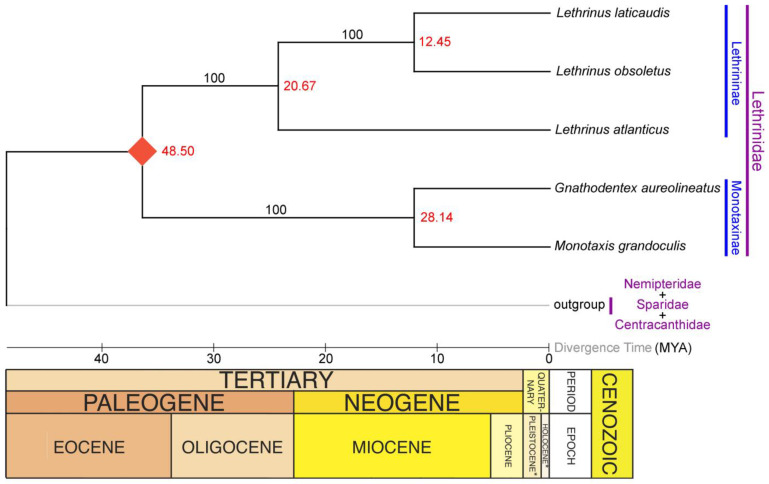
Maximum Likelihood-based TimeTree, elucidating the approximate divergence time of *L. atlanticus* from other congeners. The approximate divergence times are displayed in red, with bootstrap supports indicated at each node by black values. The red rhombus shape denotes the calibration points derived from prior research, specifically the split (48.5 Ma) between the two subfamilies (Monotaxinae and Lethrininae) within the Lethrinidae family.

Taking into account the early Eocene crown age indicating the split between Monotaxinae and Lethrininae subfamilies within the Lethrinidae family approximately 48.5 million years ago (MYA), our TimeTree analysis indicates that *L. atlanticus*, known as the Atlantic Emperor, diverged from its congeners during the late Oligocene around 20.67 MYA (Figure 5). These findings align with observed patterns of diversification in Lethrinids and other marine organisms [47,48]. However, within the Monotaxinae subfamily, the split between *Monotaxis* and *Gnathodentex* occurred during the Miocene epoch, roughly 28.14 MYA (Figure 5). This variance in divergence times compared to previous research may result from incomplete data on other species within this lineage [40,41]. It is worth noting that, while most *Lethrinus* species have broad distributions in the Red Sea and Indo-West Pacific, *L. atlanticus* is restricted solely to the Eastern Atlantic. Therefore, studying the evolution and diversification of these reef-associated fishes requires considering genetic connectivity, divergent selection, and potential demographic and ecological opportunities. Integrating ML-based TimeTree computation with marine ecological factors is crucial to understanding the evolutionary scenarios of the Atlantic Emperor and their diversification and colonization in the Eastern Atlantic.

The separation of *L. atlanticus* during the late Oligocene confronts a significant environmental change on Earth [49]. This era experienced alterations in the environment and oceanic settings, transitioning from the relatively stable warmth of the early Cenozoic to later impulsive and cooler conditions [50]. Elevated temperatures throughout this epoch led to rising sea levels through glacial ice melting and salt-water expansion [51]. Additionally, tectonic activities during the Oligocene and Miocene epochs reshaped ocean basins and seafloor topography, affecting oceanic currents and marine habitats. Consequently, reef systems differed significantly from today, with many current land areas submerged beneath shallow marine waters. This period witnessed substantial natural evolution, with the appearance of modern marine species contributing to the diverse biodiversity in new-world oceans [52]. Diversification was influenced by factors such as fluctuating oceanic temperatures, fluctuating sea levels, and the makeup of new ecological niches [53].

The speciation of *L. atlanticus* with other Indo-West Pacific congeners may have evolved due to ecological selection within the pelagic ecosystems. Historically, hydrographic and climatic circumstances in the North and South Atlantic Seas have diverged gradually due to the Coriolis effect accelerated by Earth’s rotation. In the North Atlantic, distinct oceanic gyres form due to oceanic current circulation, with the warm Gulf Stream current running away northward and the cold Canary current flowing southward, contributing to the development of the North Atlantic gyre. On the contrary, the Southern Atlantic features counterclockwise current flow, was prevailed by the anticyclonic subtropical gyre, and is confined by several major oceanic currents [54]. Despite differences in hydrography, the Red Sea and Indo-West Pacific exhibit similar characteristics due to the high saline effluence from the Red Sea to the Indian Ocean, facilitating the thriving of numerous emperor species [55]. The earlier divergence time of *L. atlanticus* compared to other *Lethrinus* species during the late Oligocene suggests a possible origin of emperors in the Atlantic Ocean, subsequently diversifying into the Red Sea and Indo-West Pacific. The oceanic currents and salinity of the Atlantic Ocean may induce the limited distribution of *L. atlanticus* to continental shelf-associated reef ecosystems in western Africa, while other *Lethrinus* species possibly evolved during the Miocene and settled in the Red Sea to the Indo-West Pacific Ocean due to the effect of the Antarctic Circumpolar Current.

### 2.8. Conservation Implication of Lethrinids

Over the last twenty years, the impacts of climate change and extreme temperature events have profoundly affected marine biodiversity and ecological functions worldwide, leading to substantial declines in fisheries’ incomes and livelihoods [56]. The dearth of comprehensive life history information for many species presents challenges in accurately assessing their vulnerability to overexploitation and the efficacy of multi-species management strategies [57,58]. In addition to the pervasive impacts of climate change, anthropogenic activities continuously pose menaces to reef fish, including Lethrinids, throughout all marine ecosystems [59,60,61,62,63]. Despite these challenges, marine fishes such as emperors remain relatively understudied on a global scale, with significant gaps in biodiversity assessment and genetic data generation [64,65]. Given the conservation importance of emperors, the presence of a single species restricted within a limited range in the Atlantic Ocean emphasizes the need for governed harvesting within the fishery management framework [66]. Adaptations in fishery management may require shifts in fishing locations, target species, and the protection of Marine Protected Areas (MPAs), spotlighting the necessity for flexible exploitation exercises and management strategies for Atlantic fish in the West African reef system. However, the known climate change projections suggest an increase in freshwater runoff in the eastern Atlantic, affecting river basins such as the Congo, Gambia, Niger, Senegal, and Volta. This anticipated rise in runoff may reduce salinity levels, potentially limiting the dispersal of several marine species. This changing scenario increases concerns about the possible local extinction of fish species associated with local reefs, seagrass beds, estuaries, and mangroves, emphasizing the imperative need for inclusive conservation measures.

## 3. Materials and Methods

### 3.1. Sampling and Species Identification

The Atlantic Emperor, *L. atlanticus*, sample was caught from the Eastern Atlantic near the coast of Cameroon, Africa (Figure 1A). Species identification was confirmed based on morphological characteristics outlined in the previous literature [67,68]. Key features include a body depth surpassing head length, jaws extending to the vertical line through the anterior or posterior nostril, a flat or slightly concave interorbital area, the third to fifth dorsal spines being the longest, the longest anal ray approximately equaling the soft anal base, five scale rows between the lateral line and median dorsal spines, an unscaled inner base of the pectoral fin, and small, pointed lateral teeth of the jaws. A sufficient amount of muscle tissue was carefully excised from the ventral thoracic region and stored in 90% ethanol for subsequent molecular experiments. A voucher specimen was stored in 10% formaldehyde at the Ministry of Livestock, Fisheries and Animal Industries (MINEPIA), Yaounde, Cameroon. The experimental protocol received approval from the IACUC (Institutional Animal Care and Use Committee) code PKNUIACUC-2022-72, ensuring compliance with ethical standards and minimizing harm to the subject fish. The global distribution range of *L. atlanticus* and related species was mapped using IUCN data (.shp files) (Figure 1A).

### 3.2. DNA Extraction and Sequencing

Genomic DNA extraction utilized the AccuPrep^®^ DNA isolation kit manufactured by Bioneer in Daejeon, Republic of Korea, adopting standard procedures. The quantity and quality of the extracted DNA were evaluated by using a NanoDrop Microvolume spectrophotometer (Thermo Fisher Scientific D1000, Waltham, MA, USA). The complete mitogenome of *L. atlanticus* underwent sequencing on the NovaSeq platform at Macrogen (https://dna.macrogen.com/) in Daejeon, Republic of Korea, employing Illumina technology. The sequencing library was developed following the TruSeq Nano DNA High-Throughput Library Prep Kit protocol (Illumina, Inc., San Diego, CA, USA). Initially, 100 ng of extracted genomic DNA was fragmented through an adaptive focused acoustic tool (Covaris, Woburn, MA, USA), generating DNA molecules with blunt ends and 5′-phosphorylation. Complying with the end-repair process, DNA fragments were selected via a bead-based method, altered by introducing a single ‘A’ base, and subsequently ligated by using TruSeq DNA UD Indexing adapters. The resulting products underwent purification and polymerase chain reaction (PCR) enrichment to yield the final library. The quantification of the library was conducted using quantitative PCR (qPCR), following the standard protocol guide (KAPA Quantification Kits for Illumina Sequencing), and quality assessment was executed using the 4200 TapeStation D1000 screentape (Agilent Technologies, Santa Clara, CA, USA). Finally, the paired-end (2 × 150 bp) sequencing was carried out by Macrogen on the NovaSeq platform (Illumina, Inc., San Diego, CA, USA).

### 3.3. Mitogenome Assembly

To trim adapters and eliminate low-quality bases, more than 20 million raw reads were screened utilizing the Cutadapt tool (https://cutadapt.readthedocs.org/, accessed on 12 June 2024) employing a Phred quality score (Q score) threshold of 20. The targeted mitogenome was assembled from high-quality paired-end reads using Geneious Prime version 2023.0.1. The reference mitogenome of *L. obsoletus* (GenBank Accession No. AP009165) was utilized with default mapping algorithms during the assembly process. Validation of the assembled mitogenome was performed by scrutinizing overlapping regions with MEGA X [69]. The boundaries and strand orientations of each gene were confirmed using MITOS Version 1.1.6 in conjunction with Galaxy Version 1.1.6 [70,71]. Additionally, validation of fish mitochondrial genome annotation was conducted using the MitoAnnotator webserver (http://mitofish.aori.u-tokyo.ac.jp/annotation/input/, accessed on 12 June 2024) [3]. To corroborate the translated amino acid sequences of each PCG, screening was conducted using the Open Reading Frame Finder web tool (https://www.ncbi.nlm.nih.gov/orffinder/, accessed on 12 June 2024), based on the vertebrate mitochondrial genetic code.

### 3.4. Validation of Control Region

To verify the entirety of the CR, a new primer pair (5′-CGTTGCAATTCTTACATGAATTGG-3′ and 5′-CCTGATACCGGCTCCTTGTC-3′) was custom-designed. PCR amplification was carried out utilizing the TaKaRa Verity Thermal Cycler, with a reaction mixture consisting of 1 U Taq polymerase, 1X PCR buffer, 10 pmol of each primer, 2.5 mM dNTPs, and 1 µL template DNA of *L. atlanticus*. Purification of the amplified product was conducted using the AccuPrep^®^ PCR/Gel Purification Kit (Bioneer, Daejeon, Republic of Korea). Subsequently, the amplicon was further amplified using the BigDye^®^ Terminator v3.1 Cycle Sequencing Kit (Applied Biosystems, Foster City, CA, USA) and bidirectionally sequenced on the ABI PRISM 3730XL DNA analyzer at Macrogen. The overlapping regions of the CR and the annotated mitogenome were aligned using MEGA X, following the removal of noisy base pairs with SeqScanner version 1.0 (Applied Biosystems Inc., Foster City, CA, USA). The final mitogenome sequence of *L. atlanticus* was deposited in GenBank to obtain an accession number.

### 3.5. Characterization and Comparative Analyses

The MitoAnnotator online tool (http://mitofish.aori.u-tokyo.ac.jp/annotation/input/, accessed on 12 August 2024) was utilized to generate the circular representation of the assembled mitogenome. This study aimed to conduct comparative analyses of the structure and variations in the *L. atlanticus* mitogenome, contrasting it with the mitogenomes of two other *Lethrinus* species: *L. obsoletus* (Accession No. AP009165) and *L. laticaudis* (Accession No. KU530221) [16,22] (Supplementary Table S7). Intergenic spacers between adjacent genes and overlapping regions were identified manually. Nucleotide compositions of PCGs, rRNAs, tRNAs, and CR were determined using MEGA X. Base composition skews were calculated using established formulas: GC-skew = [G − C]/[G + C] and AT-skew = [A − T]/[A + T] [72]. Initiation and termination codons of each PCG were confirmed using the vertebrate mitochondrial genetic code through MEGA X. The comparative analysis also included the computation of RSCU, determination of the relative abundance of amino acids, and assessment of CDsPT using DnaSP 6.0 [73]. Nonsynonymous (Ka) and synonymous (Ks) substitutions between *L. atlanticus* and other congeners were examined using DnaSP 6.0. Additionally, the gene boundaries of tRNA and rRNA genes were confirmed using the tRNAscan-SE Search Server 2.0 and ARWEN 1.2 [74,75]. Structural domains within the CR were identified and compared through CLUSTAL X alignments, as outlined in previous studies [2,76].

### 3.6. Mitogenomic Phylogenetic Analyses

To enlighten the evolutionary relationships among *Lethrinus* species, thorough phylogenetic analyses were undertaken utilizing mitochondrial genomes from 43 Spariformes species spanning diverse families. This included representatives from Lethrinidae (4 species), Centracanthidae (1 species), Nemipteridae (10 species), and Sparidae (28 species), all obtained from the GenBank database (Supplementary Table S7). *Lobotes surinamensis*’s mitochondrial genome sequence (Accession No. AB355912) from the order Lobotiformes was chosen as the outgroup taxon. For phylogenetic tree construction, a concatenated dataset comprising 13 PCGs was assembled utilizing iTaxoTools 0.1 [77]. The model selection process ‘GTR + G + I’ was performed through PartitionFinder 2 on the CIPRES Science Gateway v3.3 and JModelTest v2. This model was identified as optimal for all PCGs, with the lowest BIC (Bayesian information criterion) scores [78,79,80]. The maximum likelihood (ML) phylogenetic tree was built using the IQ-Tree web server and PhyML 3.0, incorporating 1000 bootstrap samples [81,82]. The Bayesian (BA) phylogenetic tree was generated using Mr. Bayes 3.1.2, with nst = 6, and employing one cold and three hot metropolis-coupled Markov chain Monte Carlo (MCMC) chains. The analysis was run for 10,000,000 generations, sampling trees every 100th generation, and discarding the initial 25% of samples as burn-in [83]. The ML and BA trees produced were visualized via the iTOL v4 web server (https://itol.embl.de/login.cgi, accessed on 12 June 2024) [84].

### 3.7. Divergence Time Estimation and TimeTree

The RelTime ML method was employed to calculate the divergence times of lineages within the subfamily Lethrininae and the family Lethrinidae [85]. This analysis aimed to approximate the divergence time between *L. atlanticus*, confined to the Atlantic Ocean, and two other congeners (*L. laticaudis* and *L. obsoletus*), with broad distributions from the Red Sea to the Indo-West Pacific. RelTime is known for its computational efficiency and accuracy in estimating TimeTrees, making it particularly suitable for larger datasets [86]. This approach was chosen to alleviate the substantial computational time required by BA methods [87,88]. The TimeTree was constructed using the ML phylogeny, with a single calibration constraint applied to the divergence between the two subfamilies Monotaxinae and Lethrininae (44–80 Ma). This constraint was based on one of the most well-supported nodes in emperor fishes, as corroborated by previous studies and the TimeTree of Life (https://timetree.org/, accessed on 12 June 2024) [7,40,43,89]. Upon inputting the sequence data, the ML topology (.nwk format) served as the baseline tree. After assigning the outgroup taxa, the TimeTree computation integrated calibration constraints using the calibration editor.

## 4. Conclusions

This study conducts a thorough investigation of the complete mitochondrial genome of *L. atlanticus*, elucidating its genetic configuration, structural association, and evolutionary path. Moreover, it explores the phylogenetic connections, offering insights into the evolutionary relations among *Lethrinus* species and their wider placement in the Spariformes lineage. The discussion extended to divergence timelines and diversification patterns, providing perspectives on the emergence and speciation of *L. atlanticus* in the Atlantic Sea, alongside *L. laticaudis* and *L. obsoletus* in the Red Sea and Indo-West Pacific. The intricate relationship between hydrographic factors, oceanic currents, and environmental parameters significantly influenced the evolutionary dynamics of these emperor fishes in the marine environment. Considering the possible implications for conservation, this research emphasized the importance of a comprehensive understanding of marine ecosystems and the impacts of climate change and anthropogenic activities on these reef-associated species. In summary, the current exploration of the mitochondrial genome and evolutionary chronicle of the Atlantic Emperor offers worthwhile insights into its genetic characteristics and potential environmental adaptations in the Eastern Atlantic Ocean.

## Figures and Tables

**Table 1:** List of annotated genes, including their boundaries, sizes, and intergenic nucleotides (IN) for *L. atlanticus* mitogenome. Genes placed on the negative strand in the mitogenome are highlighted in gray.

Genes	Start	Stop	Size (bp)	IN	Start Codon	Stop Codon	Anti-Codon
*tRNA-Phe (F)*	1	68	68	0			TTC
*12S rRNA*	69	1024	956	0			
*tRNA-Val (V)*	1025	1098	74	0			GTA
*16S rRNA*	1099	2899	1801	0			
*tRNA-Leu (L2)*	2900	2973	74	0			TTA
*ND1*	2974	3945	972	4	ATG	TAG	
*tRNA-Ile (I)*	3950	4019	70	−1			ATC
*tRNA-Gln (Q)*	4019	4089	71	−1			CAA
*tRNA-Met (M)*	4089	4158	70	0			ATG
*ND2*	4159	5204	1046	0	ATG	TA−	
*tRNA-Trp (W)*	5205	5277	73	0			TGA
*tRNA-Ala (A)*	5278	5346	69	1			GCA
*tRNA-Asn (N)*	5348	5420	73	37			AAC
*tRNA-Cys (C)*	5458	5526	69	0			TGC
*tRNA-Tyr (Y)*	5527	5596	70	1			TAC
*COI*	5598	7148	1551	1	GTG	TAA	
*tRNA-Ser (S2)*	7150	7220	71	3			TCA
*tRNA-Asp (D)*	7224	7295	72	7			GAC
*COII*	7303	7993	691	0	ATG	T− −	
*tRNA-Lys (K)*	7994	8068	75	1			AAA
*ATP8*	8070	8237	168	13	ATG	TAA	
*ATP6*	8251	8933	683	0	ATG	TA−	
*COIII*	8934	9718	785	0	ATG	TA−	
*tRNA-Gly (G)*	9719	9790	72	0			GGA
*ND3*	9791	10,139	349	0	ATG	T− −	
*tRNA-Arg (R)*	10,140	10,208	69	0			CGA
*ND4L*	10,209	10,505	297	−7	ATG	TAA	
*ND4*	10,499	11,879	1381	0	ATG	T− −	
*tRNA-His (H)*	11,880	11,948	69	0			CAC
*tRNA-Ser (S1)*	11,949	12,018	70	4			AGC
*tRNA-Leu (L1)*	12,023	12,095	73	0			CTA
*ND5*	12,096	13,934	1839	−4	ATG	TAA	
*ND6*	13,931	14,452	522	0	ATG	TAG	
*tRNA-Glu (E)*	14,453	14,521	69	4			GAA
*CYTB*	14,526	15,666	1141	0	ATG	T− −	
*tRNA-Thr (T)*	15,667	15,738	72	−1			ACA
*tRNA-Pro (P)*	15,738	15,806	69	0			CCA
*Control region*	15,807	16,789	983				

**Table 2:** Nucleotide composition of mitogenomes across different *Lethrinus* species.

Species Name	Size (bp)	A%	T%	G%	C%	A + T%	AT-Skew	GC-Skew
Complete mitogenome								
*L. atlanticus*	16,789	27.20	25.90	17.02	29.88	53.10	0.024	−0.274
*L. laticaudis*	16,758	26.98	25.63	17.10	30.30	52.61	0.026	−0.279
*L. obsoletus*	16,779	26.77	25.24	17.26	30.73	52.01	0.029	−0.281
Protein-coding genes (PCGs)								
*L. atlanticus*	11,425	24.7	27.8	16.4	31.1	52.5	−0.058	−0.308
*L. laticaudis*	11,423	24.5	27.4	16.6	31.6	51.9	−0.061	−0.311
*L. obsoletus*	11,425	24	26.9	16.9	32.2	51	−0.056	−0.313
Ribosomal RNAs (rRNAs)								
*L. atlanticus*	2757	30.9	21.3	21	26.8	52.3	0.184	−0.122
*L. laticaudis*	2761	30.9	21.6	21.1	26.4	52.5	0.179	−0.111
*L. obsoletus*	2664	31.4	21.7	20.7	26.2	53	0.183	−0.118
Transfer RNAs (tRNAs)								
*L. atlanticus*	1562	27.3	26.9	24.3	21.5	54.2	0.008	0.063
*L. laticaudis*	1552	27.3	27.6	24	21.1	54.9	−0.007	0.063
*L. obsoletus*	1563	27.3	26.7	24.2	21.8	54	0.012	0.051
Control Region (CR)								
*L. atlanticus*	983	31.2	30.6	14.9	23.3	61.9	0.010	−0.221
*L. laticaudis*	944	30.8	30.8	15.7	22.7	61.7	0.000	−0.182
*L. obsoletus*	970	29.7	30.2	16.9	23.2	59.9	−0.009	−0.157

## Data Availability

The complete mitogenome sequence data (Accession No. OQ420716) that endorse the findings of this research is openly accessible in GenBank of NCBI at https://www.ncbi.nlm.nih.gov.

## Supplementary Materials

The following supporting information can be downloaded at: https://www.mdpi.com/article/10.3390/ijms251910700/s1.

## References

[1] Anderson S., de Bruijn M.H., Coulson A.R., Eperon I.C., Sanger F., Young I.G. (1982). Complete sequence of bovine mitochondrial DNA. Conserved features of the mammalian mitochondrial genome. J. Mol. Biol..

[2] Satoh T.P., Miya M., Mabuchi K., Nishida M. (2016). Structure and variation of the mitochondrial genome of fishes. BMC Genom..

[3] Iwasaki W., Fukunaga T., Isagozawa R., Yamada K., Maeda Y., Satoh T.P., Sado T., Mabuchi K., Takeshima H., Miya M. (2013). MitoFish and MitoAnnotator: A mitochondrial genome database of fish with an accurate and automatic annotation pipeline. Mol. Biol. Evol..

[4] Johnson G.D. (1981). The Limits and Relationships of the Lutjanidae and Associated Families.

[5] Carpenter K.E., Allen G.R. (1989). FAO Species Catalogue. Emperor Fishes and Large-Eye Breams of the World (Family Lethrinidae). An Annotated and Illustrated Catalogue of Lethrinid Species Known to Date.

[6] Carpenter K.E., Marcus L.F., Corti M., Loy A., Naylor G.J.P., Slice D.E. (1996). Morphometric pattern and feeding mode in emperor fishes (Lethrinidae, Perciformes). Advances in Morphometrics.

[7] Lo Galbo A.M., Carpenter K.E., Reed D.L. (2002). Evolution of trophic types in emperor fishes (*Lethrinus*, Lethrinidae, Percoidei) based on cytochrome B gene sequence variation. J. Mol. Evol..

[8] Fricke R., Eschmeyer W.N., Van der Laan R. Eschmeyer’s Catalog of Fishes: Genera, Species, References. http://researcharchive.calacademy.org/research/ichthyology/catalog/fishcatmain.asp.

[9] Cuéllar-Pinzón J., Presa P., Hawkins S.J., Pita A. (2016). Genetic markers in marine fisheries: Types, tasks and trends. Fish. Res..

[10] Borsa P., Hsiao D.R., Carpenter K.E., Chen W.J. (2013). Cranial morphometrics and mitochondrial DNA sequences distinguish cryptic species of the longface emperor (*Lethrinus olivaceus*), an emblematic fish of Indo-West Pacific coral reefs. Comptes Rendus Biol..

[11] Healey A.J.E., McKeown N.J., Taylor A.L., Provan J., Sauer W., Gouws G., Shaw P.W. (2018). Cryptic species and parallel genetic structuring in Lethrinid fish: Implications for conservation and management in the southwest Indian Ocean. Ecol. Evol..

[12] Mzingirwa F.A., Stomeo F., Kaunda-Arara B., Nyunja J., Mujibi F.D.N. (2019). Genetic connectivity of the Sky Emperor, *Lethrinus mahsena* populations across a gradient of exploitation rates in coastal Kenya. Front. Genet..

[13] Afrisal M., Iwatsuki Y., Burhanuddin A.I. (2020). Morphological and genetic evaluation of the thumbprint emperor, *Lethrinus harak* (Forsskål, 1775) in the Pacific and Indian Oceans. F1000Res.

[14] Herwerden L.V., Benzie J., Peplow L., Davies C. (2000). Microsatellite markers for coral trout (*Plectropomus laevis*) and red throat emperor (*Lethrinus miniatus*) and their utility in other species of reef fish. Mol. Ecol..

[15] Miya M., Kawaguchi A., Nishida M. (2001). Mitogenomic exploration of higher teleostean phylogenies: A case study for moderate-scale evolutionary genomics with 38 newly determined complete mitochondrial DNA sequences. Mol. Biol. Evol..

[16] Yamanoue Y., Miya M., Matsuura K., Yagishita N., Mabuchi K., Sakai H., Katoh M., Nishida M. (2007). Phylogenetic position of tetraodontiform fishes within the higher teleosts: Bayesian inferences based on 44 whole mitochondrial genome sequences. Mol. Phylogenet. Evol..

[17] Al-Yousuf M.H., El-Shahawi M.S., Al-Ghais S.M. (2000). Trace metals in liver, skin, and muscle of *Lethrinus lentjan* fish species in relation to body length and sex. Sci. Total Environ..

[18] Pillans R.D., Bearham D., Boomer A., Downie R.A., Patterson T.A., Thomson D.P., Babcock R.C. (2014). Multi-year observations reveal variability in residence of a tropical demersal fish, *Lethrinus nebulosus*: Implications for spatial management. PLoS ONE.

[19] Varea R., Paris A., Ferreira M., Piovano S. (2021). Multibiomarker responses to polycyclic aromatic hydrocarbons and microplastics in thumbprint emperor *Lethrinus harak* from a South Pacific locally managed marine area. Sci. Rep..

[20] Grandcourt E.M., Al Abdessalaam T.Z., Francis F., Al Shamsi A.T. (2010). Reproductive biology and implications for management of the spangled emperor *Lethrinus nebulosus* in the southern Arabian Gulf. J. Fish Biol..

[21] Younis E.M., Abdel-Warith A.A., Al-Asgah N.A., Gabr M.H., Shamlol F.S. (2020). Demographic structure and stock status of *Lethrinus lentjan* in Saudi coastal waters of the Red Sea. Saudi J. Biol. Sci..

[22] Taillebois L., Crook D.A., Saunders T., Williams S.M., Ovenden J.R. (2016). The complete mitochondrial genome of the grass emperor, *Lethrinus laticaudis* (Perciformes: Lethrinidae). Mitochondrial DNA B Resour..

[23] Guo M., Gao Y., Huang H. (2023). The complete mitochondrial genome of striped large-eye bream, *Gnathodentex aureolineatus* (Teleostei, Lethrinidae). Mitochondrial DNA B Resour..

[24] Kundu S., De Alwis P.S., Kim A.R., Lee S.R., Kang H.-E., Go Y., Gietbong F.Z., Wibowo A., Kim H.-W. (2023). Mitogenomic characterization of Cameroonian endemic *Coptodon camerunensis* (Cichliformes: Cichlidae) and matrilineal phylogeny of Old-World cichlids. Genes.

[25] Kundu S., Kim H.-W., Lee J., Chung S., Lee S.R., Gietbong F.Z., Wibowo A., Kang K. (2023). Mitogenomic architecture and phylogenetic relationship of European barracuda, *Sphyraena sphyraena* (Teleostei: Sphyraenidae) from the Atlantic Ocean. Fishes.

[26] Ojala D., Montoya J., Attardi G. (1981). tRNA punctuation model of RNA processing in human mitochondria. Nature.

[27] Molina-Quirós J.L., Hernández-Muñoz S., Baeza J.A. (2022). The complete mitochondrial genome of the roosterfish *Nematistius pectoralis* Gill 1862: Purifying selection in protein coding genes, organization of the control region, and insights into family-level phylogenomic relationships in the recently erected order Carangiformes. Gene.

[28] Kundu S., Palimirmo F.S., Kang H.-E., Kim A.R., Lee S.R., Gietbong F.Z., Song S.H., Kim H.-W. (2023). Insights into the mitochondrial genetic makeup and Miocene colonization of primitive flatfishes (Pleuronectiformes: Psettodidae) in the East Atlantic and Indo-West Pacific Ocean. Biology.

[29] Kosiol C., Vinar T., da Fonseca R.R., Hubisz M.J., Bustamante C.D., Nielsen R., Siepel A. (2008). Patterns of positive selection in six mammalian genomes. PLoS Genet..

[30] Foote A.D., Morin P.A., Durban J.W., Pitman R.L., Wade P., Willerslev E., Gilbert M.T., da Fonseca R.R. (2011). Positive selection on the killer whale mitogenome. Biol. Lett..

[31] Yang Z.H., Nielsen R. (2000). Estimating synonymous and nonsynonymous substitution rates under realistic evolutionary models. Mol. Biol. Evol..

[32] Kundu S., Kang H.-E., Kim A.R., Lee S.R., Kim E.-B., Amin M.H.F., Andriyono S., Kim H.-W., Kang K. (2024). Mitogenomic characterization and phylogenetic placement of African hind, *Cephalopholis taeniops*: Shedding light on the evolution of groupers (Serranidae: Epinephelinae). Int. J. Mol. Sci..

[33] Zhu K.C., Liang Y.Y., Wu N., Guo H.Y., Zhang N., Jiang S.G., Zhang D.C. (2017). Sequencing and characterization of the complete mitochondrial genome of Japanese Swellshark (*Cephalloscyllium umbratile*). Sci. Rep..

[34] Crick F.H.C. (1966). Codon-anticodon pairing: The wobble hypothesis. J. Mol. Biol..

[35] Varani G., McClain W.H. (2000). The G-U wobble base pair: A fundamental building block of RNA structure crucial to RNA function in diverse biological systems. EMBO Rep..

[36] Cantatore P., Gadaleta M.N., Roberti M., Saccone C., Wilson A.C. (1987). Duplication and remoulding of tRNA genes during the evolutionary rearrangement of mitochondrial genomes. Nature.

[37] Ponce M., Infante C., Jiménez-Cantizano R.M., Pérez L., Manchado M. (2008). Complete mitochondrial genome of the blackspot seabream, *Pagellus bogaraveo* (Perciformes: Sparidae), with high levels of length heteroplasmy in the WANCY region. Gene.

[38] Lee W.J., Conroy J., Howell W.H., Kocher T.D. (1995). Structure and evolution of teleost mitochondrial control regions. J. Mol. Evol..

[39] San Mauro D., Gower D.J., Zardoya R., Wilkinson M. (2006). A hotspot of gene order rearrangement by tandem duplication and random loss in the vertebrate mitochondrial genome. Mol. Biol. Evol..

[40] Fabian V., Houk P., Lemer S. (2021). Phylogeny of Micronesian emperor fishes and evolution of trophic types. Mol. Phylogenet. Evol..

[41] Chen W.-J., Borsa P. (2020). Diversity, phylogeny, and historical biogeography of largeeye seabreams (Teleostei: Lethrinidae). Mol. Phylogenet. Evol..

[42] Tine M., Kuhl H., Gagnaire P.A., Louro B., Desmarais E., Martins R.S., Hecht J., Knaust F., Belkhir K., Klages S. (2014). European Sea bass genome and its variation provide insights into adaptation to euryhalinity and speciation. Nat. Commun..

[43] Rabosky D.L., Chang J., Title P.O., Cowman P.F., Sallan L., Friedman M., Kaschner K., Garilao C., Near T.J., Coll M. (2018). An inverse latitudinal gradient in speciation rate for marine fishes. Nature.

[44] Thi O.T., Ha Q.V.D., Thuy B.D. Phylogenetic relationships of emperors (Lethrinidae) and snappers (Lutjanidae) in Vietnam based on mitochondrial DNA sequences. Proceedings of the International Conference on Biological, Environment and Food Engineering (BEFE-2015).

[45] Waterhouse L., Heppell S.A., Pattengill-Semmens C.V., McCoy C., Bush P., Johnson B.C., Semmens B.X. (2020). Recovery of critically endangered Nassau grouper (*Epinephelus striatus*) in the Cayman Islands following targeted conservation actions. Proc. Natl. Acad. Sci. USA.

[46] Natsidis P., Tsakogiannis A., Pavlidis P., Tsigenopoulos C.S., Manousaki T. (2019). Phylogenomics investigation of sparids (Teleostei: Spariformes) using high-quality proteomes highlights the importance of taxon sampling. Commun. Biol..

[47] Alfaro M.E., Santini F., Brock C.D. (2007). Do reefs drive diversification in marine teleosts? Evidence from the pufferfish and their allies (Order Tetraodontiformes). Evolution.

[48] Alfaro M.E., Santini F., Brock C., Alamillo H., Dornburg A., Rabosky D.L., Carnelave G., Hamon L.J. (2009). Nine exceptional radiations plus high turnover explain species diversity in jawed vertebrates. Proc. Natl. Acad. Sci. USA.

[49] Ao H., Rohling E.J., Zhang R., Roberts A.P., Holbourn A.E., Ladant J.B., Dupont-Nivet G., Kuhnt W., Zhang P., Wu F. (2021). Global warming-induced Asian hydrological climate transition across the Miocene–Pliocene boundary. Nat. Commun..

[50] Shevenell A.E., Kennett J.P., Lea D.W. (2004). Middle Miocene Southern Ocean cooling and Antarctic cryosphere expansion. Science.

[51] Methner K., Campani M., Fiebig J., Löffler N., Kempf O., Mulch A. (2020). Middle Miocene long-term continental temperature change in and out of pace with marine climate records. Sci. Rep..

[52] Herbert T.D., Lawrence K.T., Tzanova A., Peterson L.C., Caballero-Gill R., Kelly C.S. (2016). Late Miocene global cooling and the rise of modern ecosystems. Nat. Geosci..

[53] Avaria-Llautureo J., Venditti C., Rivadeneira M.M., Inostroza-Michael O., Rivera R.J., Hernández C.E., Canales-Aguirre C.B. (2021). Historical warming consistently decreased size, dispersal and speciation rate of fish. Nat. Clim. Chang..

[54] O’Brien T.D., Lorenzoni L., Isensee K., Valdés L. (2017). What are Marine Ecological Time Series telling us about the ocean?. A Status Report.

[55] Momigliano P., Jokinen H., Fraimout A., Florin A.B., Norkko A., Merilä J. (2017). Extraordinarily rapid speciation in a marine fish. Proc. Natl. Acad. Sci. USA.

[56] Cheung W.W.L., Frölicher T.L., Lam V.W.Y., Oyinlola M.A., Reygondeau G., Sumaila U.R., Tai T.C., The L.C.L., Wabnitz C.C.C. (2021). Marine high temperature extremes amplify the impacts of climate change on fish and fisheries. Sci. Adv..

[57] Brander K.M. (2007). Global fish production and climate change. Proc. Natl. Acad. Sci. USA.

[58] Barbarossa V., Bosmans J., Wanders N., King H., Bierkens M.F.P., Huijbregts M.A.J., Schipper A.M. (2021). Threats of global warming to the world’s freshwater fishes. Nat. Commun..

[59] Jones G.P., McCormick M.I., Srinivasan M., Eagle J.V. (2004). Coral decline threatens fish biodiversity in marine reserves. Proc. Natl. Acad. Sci. USA.

[60] Graham N.A., Chabanet P., Evans R.D., Jennings S., Letourneur Y., MacNeil M.A., McClanahan T.R., Ohman M.C., Polunin N.V., Wilson S.K. (2011). Extinction vulnerability of coral reef fishes. Ecol. Lett..

[61] MacNeil M.A., Graham N.A., Cinner J.E., Wilson S.K., Williams I.D., Maina J., Newman S., Friedlander A.M., Jupiter S., Polunin N.V. (2015). Recovery potential of the world’s coral reef fishes. Nature.

[62] Mellin C., Mouillot D., Kulbicki M., McClanahan T.R., Vigliola L., Bradshaw C.J., Brainard R.E., Chabanet P., Edgar G.J., Fordham D.A. (2016). Humans and seasonal climate variability threaten large-bodied coral reef fish with small ranges. Nat. Commun..

[63] Filous A., Daxboeck C., Beguet T., Cook C. (2022). The life history of longnose emperors (*Lethrinus olivaceus*) and a data-limited assessment of their stock to support fisheries management at Rangiroa Atoll, French Polynesia. J. Fish Biol..

[64] Hutchings J.A. (2000). Collapse and recovery of marine fishes. Nature.

[65] Costello M.J., Coll M., Danovaro R., Halpin P., Ojaveer H., Miloslavich P. (2010). A Census of Marine Biodiversity Knowledge, Resources, and Future Challenges. PLoS ONE.

[66] Currey L.M., Williams A.J., Mapstone B.D., Davies C.R., Carlos G., Welch D.J., Simpfendorfer C.A., Ballagh A.C., Penny A.L., Grandcourt E.M. (2013). Comparative biology of tropical *Lethrinus* species (Lethrinidae): Challenges for multi-species management. J. Fish Biol..

[67] Sato T. (1978). A synopsis of the sparoid fish genus *Lethrinus*, with the description of a new species. Bull. Univ. Mus. Univ. Tokyo.

[68] Carpenter K.E., De Angelis N. (2016). The Living Marine Resources of the Eastern Central Atlantic. Vol. 4: Bony Fishes Part 2 (Perciformes to Tetradontiformes) and Sea Turtles.

[69] Kumar S., Stecher G., Li M., Knyaz C., Tamura K. (2018). MEGA X: Molecular Evolutionary Genetics Analysis across computing platforms. Mol. Biol. Evol..

[70] Bernt M., Donath A., Jühling F., Externbrink F., Florentz C., Fritzsch G., Pütz J., Middendorf M., Stadler P.F. (2013). MITOS: Improved de novo Metazoan Mitochondrial Genome Annotation. Mol. Phylogenet. Evol..

[71] Blankenberg D., Von Kuster G., Bouvier E., Baker D., Afgan E., Stoler N., Taylor J., Nekrutenko A., Galaxy Team (2014). Dissemination of scientific software with Galaxy ToolShed. Genome Biol..

[72] Perna N.T., Kocher T.D. (1995). Patterns of nucleotide composition at fourfold degenerate sites of animal mitochondrial genomes. J. Mol. Evol..

[73] Rozas J., Ferrer-Mata A., Sánchez-DelBarrio J.C., Guirao-Rico S., Librado P., Ramos-Onsins S.E., Sánchez-Gracia A. (2017). DnaSP 6: DNA sequence polymorphism analysis of large data sets. Mol. Biol. Evol..

[74] Laslett D., Canbäck B. (2008). ARWEN, a program to detect tRNA genes in metazoan mitochondrial nucleotide sequences. Bioinformatics.

[75] Chan P.P., Lin B.Y., Mak A.J., Lowe T.M. (2021). tRNAscan-SE 2.0: Improved detection and functional classification of transfer RNA genes. Nucleic Acids Res..

[76] Thompson J.D., Gibson T.J., Plewniak F., Jeanmougin F., Higgins D.G. (1997). The CLUSTAL_X windows interface: Flexible strategies for multiple sequence alignment aided by quality analysis tools. Nucleic Acids Res..

[77] Vences M., Miralles A., Brouillet S., Ducasse J., Fedosov A., Kharchev V., Kostadinov I., Kumari S., Patmanidis S., Scherz M.D. (2021). iTaxoTools 0.1: Kickstarting a specimen-based software toolkit for taxonomists. Megataxa.

[78] Darriba D., Taboada G.L., Doallo R., Posada D. (2012). JModelTest 2: More models, new heuristics and parallel computing. Nat. Methods.

[79] Miller M.A., Schwartz T., Pickett B.E., He S., Klem E.B., Scheuermann R.H., Passarotti M., Kaufman S., O’Leary M.A. (2015). A RESTful API for Access to Phylogenetic Tools via the CIPRES Science Gateway. Evol. Bioinform..

[80] Lanfear R., Frandsen P.B., Wright A.M., Senfeld T., Calcott B. (2016). PartitionFinder 2: New Methods for Selecting Partitioned Models of Evolution for Molecular and Morphological Phylogenetic Analyses. Mol. Biol. Evol..

[81] Trifinopoulos J., Nguyen L.-T., von Haeseler A., Minh B.Q. (2016). W-IQ-TREE: A fast online phylogenetic tool for maximum likelihood analysis. Nucleic Acids Res..

[82] Guindon S., Dufayard J.F., Lefort V., Anisimova M., Hordijk W., Gascuel O. (2010). New Algorithms and Methods to Estimate Maximum-Likelihood Phylogenies: Assessing the Performance of PhyML 3.0. Syst. Biol..

[83] Ronquist F., Huelsenbeck J.P. (2003). MrBayes 3: Bayesian phylogenetic inference under mixed models. Bioinformatics.

[84] Letunic I., Bork P. (2007). Interactive Tree of Life (iTOL): An online tool for phylogenetic tree display and annotation. Bioinformatics.

[85] Mello B. (2018). Estimating TimeTrees with MEGA and the TimeTree Resource. Mol. Biol. Evol..

[86] Tamura K., Tao Q., Kumar S., Russo C. (2018). Theoretical foundation of the reltime method for estimating divergence times from variable evolutionary rates. Mol. Biol. Evol..

[87] Tamura K., Battistuzzi F.U., Billing-Ross P., Murillo O., Filipski A., Kumar S. (2012). Estimating divergence times in large molecular phylogenies. Proc. Natl. Acad. Sci. USA.

[88] Mello B., Tao Q., Tamura K., Kumar S. (2017). Fast and Accurate Estimates of Divergence Times from Big Data. Mol. Biol. Evol..

[89] Kumar S., Stecher G., Suleski M., Hedges S.B. (2017). Timetree: A resource for timelines, timetrees, and divergence times. Mol. Biol. Evol..

