# Supplementary material for: Mitogenomic Architecture of Atlantic Emperor Lethrinus atlanticus (Actinopterygii: Spariformes): Insights into the Lineage Diversification in Atlantic Ocean
Source: Int J Mol Sci. 2024 Oct 4;25(19):10700. doi: 10.3390/ijms251910700 (PMC11476654; doi:10.3390/ijms251910700)
Supplement: Supplementary file 1 [file ijms-25-10700-s001.zip › ijms-3245594-supplementary.pdf]

Supplementary Material

Table S1. Intergenic nucleotides and overlapping regions of different genes of *Lethrinus* species mitogenomes.

Genes	*Lethrinus atlanticus*	*Lethrinus laticaudis*	*Lethrinus obsoletus*
tRNA-Phe (F)	0	0	0
12S rRNA	0	-1	0
tRNA-Val (V)	0	-2	96
16S rRNA	0	0	0
tRNA-Leu (L2)	0	-1	0
ND1	4	4	4
tRNA-Ile (I)	-1	-1	-1
tRNA-Gln (Q)	-1	-2	-1
tRNA-Met (M)	0	-1	0
ND2	0	2	0
tRNA-Trp (W)	0	-1	0
tRNA-Ala (A)	1	2	1
tRNA-Asn (N)	37	37	37
tRNA-Cys (C)	0	1	-1
tRNA-Tyr (Y)	1	8	1
COI	1	2	1
tRNA-Ser (S2)	3	3	3
tRNA-Asp (D)	7	7	7
COII	0	0	0
tRNA-Lys (K)	1	0	1
ATP8	13	12	13
ATP6	0	-1	0
COIII	0	0	0
tRNA-Gly (G)	0	0	0
ND3	0	0	0
tRNA-Arg (R)	0	2	1
ND4L	-7	-7	-7
ND4	0	0	0
tRNA-His (H)	0	1	0
tRNA-Ser (S1)	4	16	3
tRNA-Leu (L1)	0	0	0
ND5	-4	-4	-4
ND6	0	0	0
tRNA-Glu (E)	4	4	4
CYTB	0	0	0
tRNA-Thr (T)	-1	-2	-1
tRNA-Pro (P)	0	0	0
Control region	–	–	–

Table S2. Comprehensive comparison of the start and stop codons of the PCGs of *Lethrinus* mitogenomes.

PCGs	*Lethrinus atlanticus*		*Lethrinus laticaudis*		*Lethrinus obsoletus*	
	**Start**	**Stop**	**Start**	**Stop**	**Start**	**Stop**
ND1	ATG	TAG	ATG	TAG	ATG	TAA
ND2	ATG	TA–	ATG	TA–	ATG	TA–
COI	GTG	TAA	ATT	TAA	GTG	TAA
COII	ATG	T– –	ATG	T– –	ATG	T– –
ATP8	ATG	TAA	ATG	TAA	ATG	TAA
ATP6	ATG	TA–	ATG	TA–	ATG	TA–
COIII	ATG	TA–	ATG	TA–	ATG	TA–
ND3	ATG	T– –	ATG	T– –	ATG	T– –
ND4L	ATG	TAA	ATG	TAA	ATG	TAA
ND4	ATG	T– –	ATG	T– –	ATG	T– –
ND5	ATG	TAA	ATG	TAA	ATG	TAG
ND6	ATG	TAG	ATG	TAG	ATG	TAA
CYTB	ATG	T– –	ATG	T– –	ATG	T– –

Table S3. Relative Synonymous Codon Usage (RSCU) value of the complete PCGs of *Lethrinus* mitogenomes.

Lathrinus atlanticus											
Codon	Count	RSCU	Codon	Count	RSCU	Codon	Count	RSCU	Codon	Count	RSCU
UUU(F)	88	0.84	UCU(S)	58	1.13	UAU(Y)	45	0.74	UGU(C)	18	0.72
UUC(F)	121	1.16	UCC(S)	88	1.72	UAC(Y)	76	1.26	UGC(C)	32	1.28
UUA(L)	87	0.88	UCA(S)	63	1.23	UAA(*)	37	1.7	UGA(W)	77	1.39
UUG(L)	35	0.35	UCG(S)	19	0.37	UAG(*)	16	0.74	UGG(W)	34	0.61
CUU(L)	143	1.45	CCU(P)	106	1.37	CAU(H)	48	0.77	CGU(R)	25	0.95
CUC(L)	144	1.46	CCC(P)	111	1.43	CAC(H)	76	1.23	CGC(R)	25	0.95
CUA(L)	131	1.33	CCA(P)	67	0.86	CAA(Q)	84	1.5	CGA(R)	33	1.26
CUG(L)	53	0.54	CCG(P)	26	0.34	CAG(Q)	28	0.5	CGG(R)	22	0.84
AUU(I)	106	1	ACU(T)	54	0.76	AAU(N)	54	0.79	AGU(S)	30	0.59
AUC(I)	106	1	ACC(T)	112	1.58	AAC(N)	82	1.21	AGC(S)	49	0.96
AUA(M)	58	0.83	ACA(T)	89	1.26	AAA(K)	69	1.6	AGA(*)	13	0.6
AUG(M)	81	1.17	ACG(T)	28	0.4	AAG(K)	17	0.4	AGG(*)	21	0.97
GUU(V)	43	0.99	GCU(A)	57	0.79	GAU(D)	33	0.81	GGU(G)	37	0.8
GUC(V)	54	1.24	GCC(A)	137	1.9	GAC(D)	48	1.19	GGC(G)	58	1.25
GUA(V)	52	1.2	GCA(A)	81	1.13	GAA(E)	56	1.24	GGA(G)	48	1.04
GUG(V)	25	0.57	GCG(A)	13	0.18	GAG(E)	34	0.76	GGG(G)	42	0.91
Lethrinus laticaudis											
Codon	Count	RSCU	Codon	Count	RSCU	Codon	Count	RSCU	Codon	Count	RSCU
UUU(F)	81	0.84	UCU(S)	70	1.27	UAU(Y)	50	0.84	UGU(C)	16	0.64
UUC(F)	111	1.16	UCC(S)	87	1.58	UAC(Y)	69	1.16	UGC(C)	34	1.36
UUA(L)	71	0.71	UCA(S)	64	1.16	UAA(*)	39	1.54	UGA(W)	65	1.37
UUG(L)	28	0.28	UCG(S)	24	0.44	UAG(*)	18	0.71	UGG(W)	30	0.63
CUU(L)	140	1.4	CCU(P)	119	1.47	CAU(H)	43	0.77	CGU(R)	22	0.86
CUC(L)	154	1.54	CCC(P)	117	1.44	CAC(H)	69	1.23	CGC(R)	21	0.82
CUA(L)	136	1.36	CCA(P)	67	0.83	CAA(Q)	79	1.4	CGA(R)	36	1.41
CUG(L)	72	0.72	CCG(P)	21	0.26	CAG(Q)	34	0.6	CGG(R)	23	0.9
AUU(I)	117	1.1	ACU(T)	47	0.71	AAU(N)	62	0.91	AGU(S)	29	0.53
AUC(I)	96	0.9	ACC(T)	114	1.71	AAC(N)	74	1.09	AGC(S)	56	1.02
AUA(M)	63	0.89	ACA(T)	87	1.31	AAA(K)	72	1.73	AGA(*)	19	0.75
AUG(M)	79	1.11	ACG(T)	18	0.27	AAG(K)	11	0.27	AGG(*)	25	0.99
GUU(V)	47	1.13	GCU(A)	69	0.9	GAU(D)	25	0.66	GGU(G)	23	0.5
GUC(V)	57	1.37	GCC(A)	137	1.79	GAC(D)	51	1.34	GGC(G)	69	1.49
GUA(V)	40	0.96	GCA(A)	80	1.04	GAA(E)	54	1.2	GGA(G)	42	0.91
GUG(V)	23	0.55	GCG(A)	21	0.27	GAG(E)	36	0.8	GGG(G)	51	1.1
Lethrinus obsoletus											
Codon	Count	RSCU	Codon	Count	RSCU	Codon	Count	RSCU	Codon	Count	RSCU
UUU(F)	80	0.82	UCU(S)	67	1.29	UAU(Y)	54	0.96	UGU(C)	16	0.65
UUC(F)	114	1.18	UCC(S)	91	1.75	UAC(Y)	59	1.04	UGC(C)	33	1.35
UUA(L)	68	0.68	UCA(S)	51	0.98	UAA(*)	39	0.93	UGA(*)	66	1.57
UUG(L)	34	0.34	UCG(S)	24	0.46	UAG(*)	21	0.5	UGG(W)	28	1
CUU(L)	135	1.36	CCU(P)	118	1.43	CAU(H)	34	0.59	CGU(R)	18	0.72

CUC(L)	159	1.6	CCC(P)	121	1.47	CAC(H)	82	1.41	CGC(R)	30	1.21
CUA(L)	129	1.3	CCA(P)	64	0.78	CAA(Q)	85	1.49	CGA(R)	39	1.57
CUG(L)	72	0.72	CCG(P)	26	0.32	CAG(Q)	29	0.51	CGG(R)	27	1.09
AUU(I)	92	1.04	ACU(T)	41	0.62	AAU(N)	59	0.8	AGU(S)	32	0.62
AUC(I)	116	1.31	ACC(T)	110	1.66	AAC(N)	88	1.2	AGC(S)	47	0.9
AUA(I)	58	0.65	ACA(T)	91	1.37	AAA(K)	67	1.58	AGA(R)	15	0.6
AUG(M)	78	1	ACG(T)	23	0.35	AAG(K)	18	0.42	AGG(R)	20	0.81
GUU(V)	54	1.22	GCU(A)	70	0.89	GAU(D)	20	0.56	GGU(G)	22	0.47
GUC(V)	56	1.27	GCC(A)	141	1.8	GAC(D)	52	1.44	GGC(G)	70	1.48
GUA(V)	36	0.81	GCA(A)	84	1.07	GAA(E)	47	1.13	GGA(G)	55	1.16
GUG(V)	31	0.7	GCG(A)	19	0.24	GAG(E)	36	0.87	GGG(G)	42	0.89

Table S4. RSCU Abundance and codons per thousand codons (CDsPT) of the complete PCGs of *Lethrinus* mitogenomes.

Amino Acids	***Lethrinus atlanticus***		***Lethrinus laticaudis***		***Lethrinus obsoletus***	
	Abundance	CDsPT	Abundance	CDsPT	Abundance	CDsPT
Ala	288	75.72968709	307	80.70452156	314	82.56639495
Arg	105	27.60978175	102	26.81388013	114	29.97633447
Asn	136	35.76124113	136	35.75184017	147	38.65369445
Asp	81	21.29897449	76	19.97896951	72	18.93242177
Cys	50	13.14751512	50	13.14405889	49	12.88456482
Gln	112	29.45043387	113	29.70557308	114	29.97633447
Glu	90	23.66552722	90	23.65930599	83	21.8248751
Gly	185	48.64580594	185	48.63301788	189	49.69760715
His	124	32.6058375	112	29.4426919	116	30.50223508
Ile	212	55.74546411	213	55.99369085	208	54.6936629
Leu	593	155.9295293	601	157.9915878	597	156.9813305
Lys	86	22.61372601	83	21.81913775	85	22.3507757
Met	139	36.55009203	142	37.32912723	136	35.76124113
Phe	209	54.9566132	192	50.47318612	194	51.01235866
Pro	310	81.51459374	324	85.17350158	329	86.51064949
Ser	307	80.72574283	330	86.75078864	312	82.04049435
Thr	283	74.41493558	266	69.92639327	265	69.68183013
Trp	111	29.18748357	95	24.97371188	94	24.71732842
Tyr	121	31.81698659	119	31.28286015	113	29.71338417
Val	174	45.75335262	167	43.90115668	177	46.54220352
Stp	87	22.87667631	101	26.55099895	95	24.98027873

Table S5. Pairwise Ka/Ks ratios of 13 PCGs in *Lethrinus* species mitogenomes.

**Gene**	***nad1***	***nad2***	***cox1***	***cox2***	***atp8***	***atp6***	***cox3***	***nad3***	***nad4l***	***nad4***	***nad5***	***nad6***	***Cytb***
	0.0128	0.0284	0.0027	0.0089	0.2209	0.0346	0.0080	0.0112	0.0302	0.0330	0.0249	0.0877	0.0107
	0.0209	0.0259	0.0029	0.0050	0.1143	0.0344	0.0079	0.0139	0.0230	0.0365	0.0214	0.0759	0.0106
	0.0273	0.0264	0.0033	0.0223	0.4637	0.0636	0.0188	0.0324	0.0086	0.0253	0.0359	0.0290	0.0078
Average	0.0203	0.0269	0.0030	0.0120	0.2663	0.0442	0.0115	0.0192	0.0206	0.0316	0.0274	0.0642	0.0097
STDEV	0.0072	0.0013	0.0003	0.0091	0.1791	0.0168	0.0063	0.0115	0.0110	0.0058	0.0076	0.0311	0.0017

Table S6. Comparison of anticodons found in the transfer RNA genes within the *Lethrinus* species mitogenomes.

**tRNAs**	***Lethrinus atlanticus***	***Lethrinus laticaudis***	***Lethrinus obsoletus***
tRNA-Phe (F)	TTC	TTC	TTC
tRNA-Val (V)	GTA	GTA	GTA
tRNA-Leu (L2)	TTA	TTA	TTA
tRNA-Ile (I)	ATC	ATC	ATC
tRNA-Gln (Q)	CAA	CAA	CAA
tRNA-Met (M)	ATG	ATG	ATG
tRNA-Trp (W)	TGA	TGA	TGA
tRNA-Ala (A)	GCA	GCA	GCA
tRNA-Asn (N)	AAC	AAC	AAC
tRNA-Cys (C)	TGC	TGC	TGC
tRNA-Tyr (Y)	TAC	TAC	TAC
tRNA-Ser (S2)	TCA	TCA	TCA
tRNA-Asp (D)	GAC	GAC	GAC
tRNA-Lys (K)	AAA	AAA	AAA
tRNA-Gly (G)	GGA	GGA	GGA
tRNA-Arg (R)	CGA	CGA	CGA
tRNA-His (H)	CAC	CAC	CAC
tRNA-Ser (S1)	AGC	AGC	AGC
tRNA-Leu (L1)	CTA	CTA	CTA
tRNA-Glu (E)	GAA	GAA	GAA
tRNA-Thr (T)	ACA	ACA	ACA
tRNA-Pro (P)	CCA	CCA	CCA

Table S7. Mitogenomes of Spariformes species used for phylogenetic analyses. Star marks represent the taxa used for TimeTree analyses.

Sl. No.	Family	Subfamily	Species	Accession Numbers
1	Lethrinidae	Lathrininae	*Lathrinus atlanticus**	OQ420716
2	Lethrinidae	Lathrininae	*Lethrinus obsoletus**	AP009165
3	Lethrinidae	Lathrininae	*Lethrinus laticaudis**	KU530221
4	Lethrinidae	Monotaxinae	*Gnathodentex aureolineatus**	OM302214
5	Lethrinidae	Monotaxinae	*Monotaxis grandoculis**	AP009166
6	Nemipteridae		*Pentapodus setosus*	LC557138
7	Nemipteridae		*Scolopsis vosmeri**	KT692978
8	Nemipteridae		*Scolopsis ciliata*	MH995531
9	Nemipteridae		*Nemipterus furcosus*	LC549804
10	Nemipteridae		*Nemipterus balinensoides*	OR546136
11	Nemipteridae		*Nemipterus hexodon*	MK978155
12	Nemipteridae		*Nemipterus randalli*	MT795184
13	Nemipteridae		*Nemipterus bathybius*	AB355917
14	Nemipteridae		*Nemipterus virgatus*	KU933270
15	Nemipteridae		*Nemipterus japonicus*	KJ473717
16	Sparidae		*Dentex angolensis*	MH593823
17	Sparidae		*Dentex tumifrons*	NC_029479
18	Sparidae		*Evynnis tumifrons*	KT724963
19	Sparidae		*Dentex hypselosomus*	MK978157
20	Sparidae		*Polysteganus undulosus*	OR609365
21	Sparidae		*Argyrops bleekeri*	MZ892909
22	Sparidae		*Parargyrops edita*	EF107158
23	Sparidae		*Pagrus major**	AP002949
24	Sparidae		*Pagellus erythrinus*	MG653592
25	Sparidae		*Pagrus pagrus*	OQ190459
26	Sparidae		*Pagrus auriga*	AB124801
27	Sparidae		*Dentex dentex*	MG727892
28	Sparidae		*Dentex gibbosus*	MG653593
29	Sparidae		*Stenotomus chrysops*	OR582685
30	Sparidae		*Lagodon rhomboides*	OP057024
31	Sparidae		*Archosargus rhomboidalis*	OP035230
32	Sparidae		*Calamus calamus*	OP056932
33	Sparidae		*Calamus penna*	OR546139
34	Sparidae		*Pagellus acarne*	MG736083
35	Sparidae		*Pagellus bogaraveo*	AB305023
36	Sparidae		*Acanthopagrus latus*	MN909968
37	Sparidae		*Acanthopagrus pacificus*	LC707238
38	Sparidae		*Acanthopagrus schlegelii*	JQ746035
39	Sparidae		*Rhabdosargus sarba*	KM272585
40	Sparidae		*Sparus aurata*	LK022698
41	Sparidae		*Diplodus cervinus*	ON417691
42	Sparidae		*Diplodus sargus*	MW559786
43	Sparidae		*Diplodus holbrookii*	OR546138
44	Centracanthidae		*Spicara maena**	AP009164

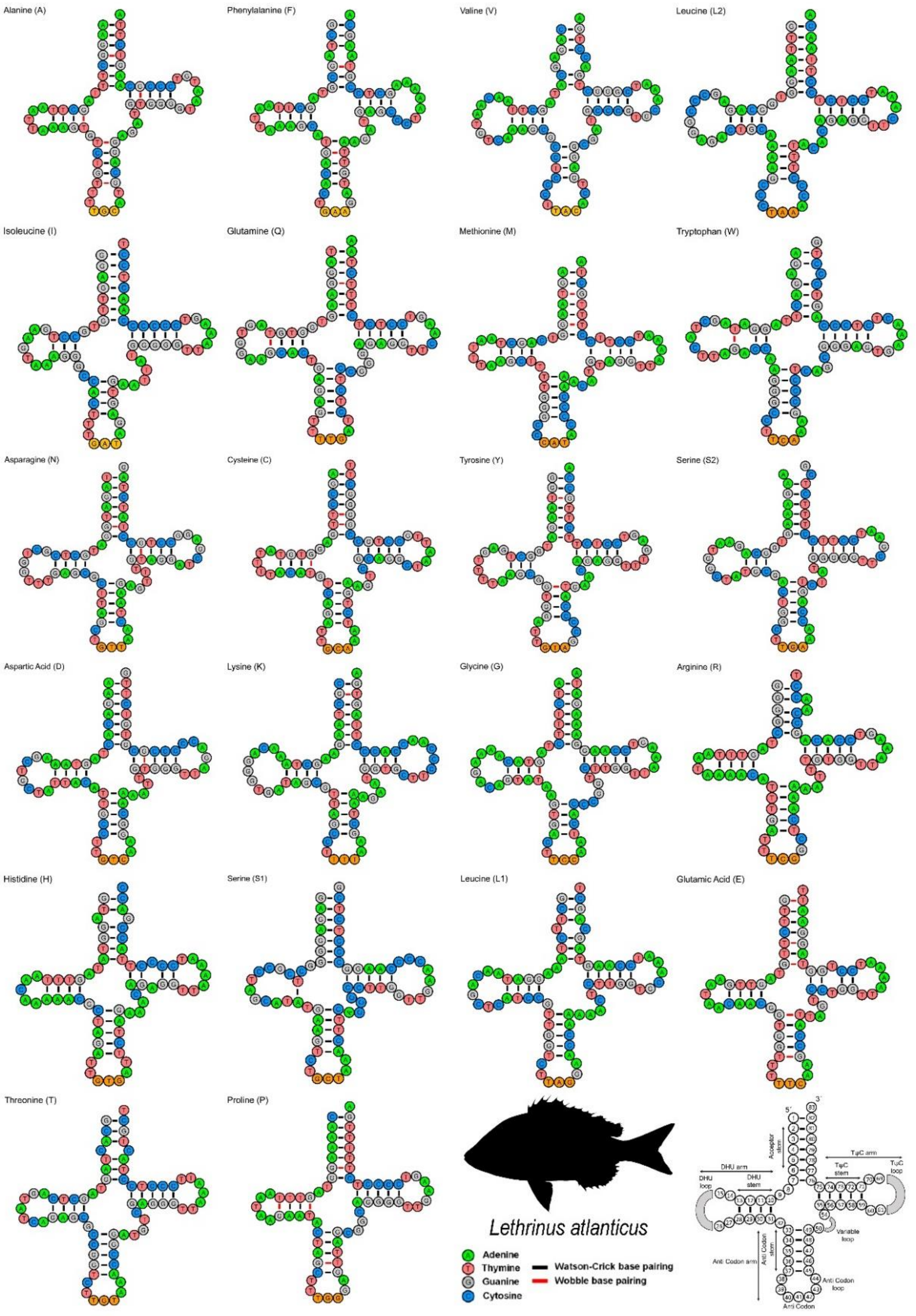

Figure S1. The secondary structures of 22 transfer RNAs (tRNAs) in the *L. atlanticus* mitogenome exhibit structural variations. These tRNAs are labeled with their complete names and single-letter amino acid codes following the IUPAC-IUB convention. The last structure provides information on the nucleotide positions and specifics of the tRNAs' stem-loop configuration.

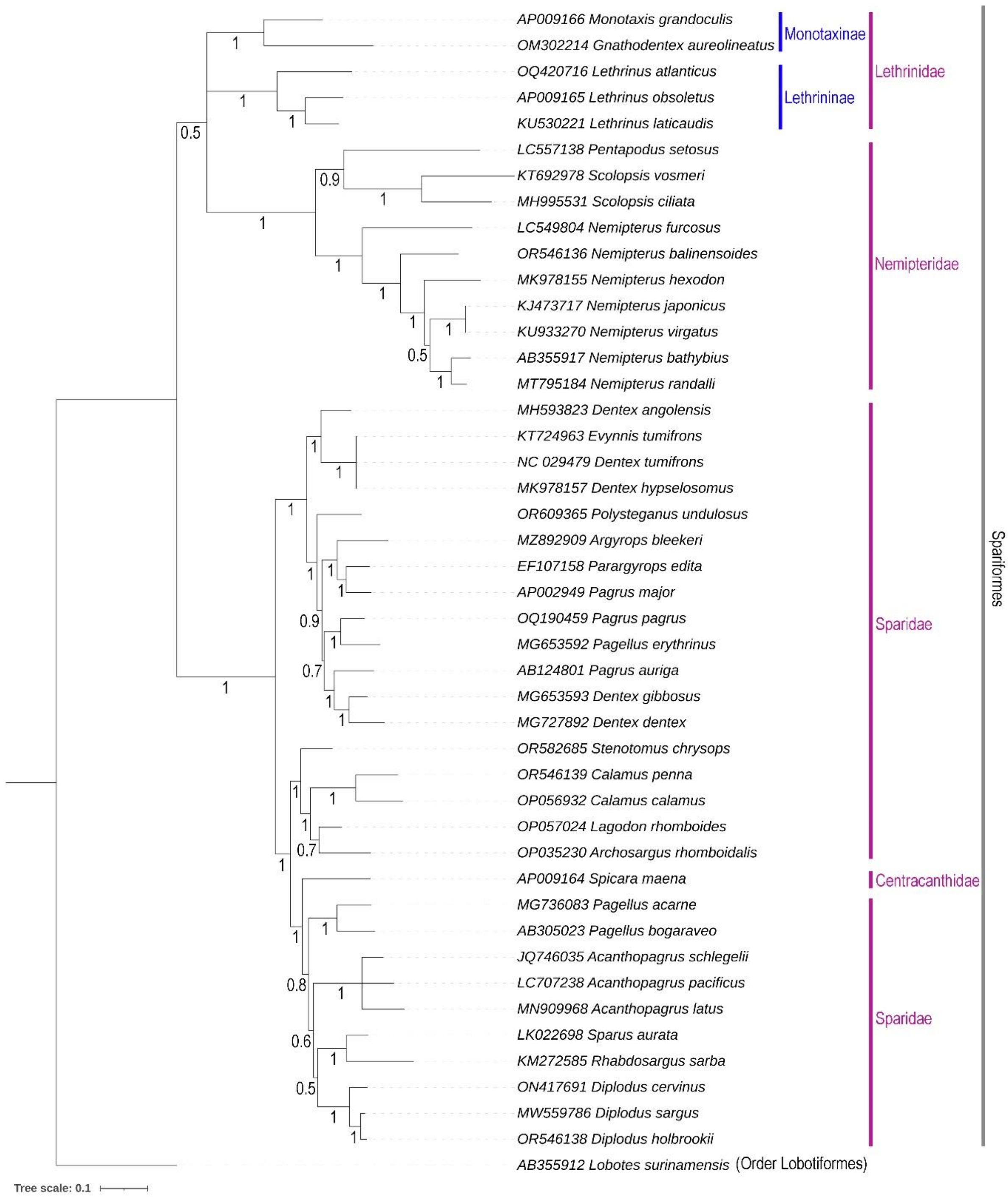

Figure S2. The Bayesian phylogeny constructed by 13 concatenated PCGs clearly discriminate *L. atlanticus* from other *Lethrinus* congeners. BA posterior probability supports are indicated at each node (black values), reflecting the statistical support for each branching point in the tree.
